# Timing is everything: early degradation of abscission layer is associated with increased seed shattering in U.S. weedy rice

**DOI:** 10.1186/1471-2229-11-14

**Published:** 2011-01-14

**Authors:** Carrie S Thurber, Peter K Hepler, Ana L Caicedo

**Affiliations:** 1 Biology Department, University of Massachusetts, Amherst, MA 01003, USA

## Abstract

**Background:**

Seed shattering, or shedding, is an important fitness trait for wild and weedy grasses. U.S. weedy rice (*Oryza sativa*) is a highly shattering weed, thought to have evolved from non-shattering cultivated ancestors. All U.S. weedy rice individuals examined to date contain a mutation in the *sh4 *locus associated with loss of shattering during rice domestication. Weedy individuals also share the shattering trait with wild rice, but not the ancestral shattering mutation at *sh4; *thus, how weedy rice reacquired the shattering phenotype is unknown. To establish the morphological basis of the parallel evolution of seed shattering in weedy rice and wild, we examined the abscission layer at the flower-pedicel junction in weedy individuals in comparison with wild and cultivated relatives.

**Results:**

Consistent with previous work, shattering wild rice individuals possess clear, defined abscission layers at flowering, whereas non-shattering cultivated rice individuals do not. Shattering weedy rice from two separately evolved populations in the U.S. (SH and BHA) show patterns of abscission layer formation and degradation distinct from wild rice. Prior to flowering, the abscission layer has formed in all weedy individuals and by flowering it is already degrading. In contrast, wild *O. rufipogon *abscission layers have been shown not to degrade until after flowering has occurred.

**Conclusions:**

Seed shattering in weedy rice involves the formation and degradation of an abscission layer in the flower-pedicel junction, as in wild *Oryza*, but is a developmentally different process from shattering in wild rice. Weedy rice abscission layers appear to break down earlier than wild abscission layers. The timing of weedy abscission layer degradation suggests that unidentified regulatory genes may play a critical role in the reacquisition of shattering in weedy rice, and sheds light on the morphological basis of parallel evolution for shattering in weedy and wild rice.

## Background

Abscission is the process by which plants shed unwanted organs, such as those that have been damaged or diseased, or release ripe seeds and fruits [1]. Seed abscission is an important mechanism for seed dispersal in many wild cereals [2]. During domestication of grass species (e.g. wheat, rye, barley, and rice), a critical shift occurred towards reductions in seed-shedding ability, facilitating the harvesting of grains [2-5]. Seed shattering is costly to farmers, as crop yield is diminished, and lost seeds may lead to persistence of crop volunteers in cultivated fields [5,6]. However, seeds that require intense labor to harvest are also undesirable, along with those that remain on the plant and germinate (i.e. preharvest sprouting). A balance between ease of shattering and difficult threshing is maintained in crop species, determined in part by specific demands of the harvesting system (e.g. hand vs. machine threshing) [7,8]. In contrast, in agricultural weeds -- plants that invade cultivated fields -- increased seed dispersal is believed to be favored, much as it is in wild species [2]. Seed shattering is a commonly observed trait in agricultural weedy plants that are related to domesticated species [2]. Seed shattering is thus under opposing selection in crops and weeds inhabiting agricultural complexes.

Domesticated Asian rice (*Oryza sativa *L.) is one of the world's most important crop species, providing about 20% of the world's caloric intake [9]. Cultivated rice fields worldwide are invaded by a weedy relative of rice known as weedy or red rice (*O. sativa*) [10]. Weedy rice is costly to farmers in terms of yield losses and removal efforts, as it competes aggressively with cultivated rice and can contaminate harvests [10,11]. The ability of weedy rice to survive and spread in cultivated rice fields has been attributed in part to its reported capacity to shatter seeds (e.g. [12-15]). High levels of seed shattering are also prevalent in the wild ancestor of cultivated rice, *O. rufipogon*, which is native to tropical wetlands of South Asia [16]. Cultivated Asian rice, in contrast, shows a wide range of seed threshability levels, from nearly shattering to difficult to thresh, but is generally less shattering than wild and weedy species [17,18].

Organ abscission in plants depends on the formation of abscission zones, which are morphologically distinct structures generally consisting of one to multiple layers of cells dense with cytoplasm [1,6]. Swelling and dissolving of the middle lamella between adjacent cell walls in the abscission layer allows for organ release [1,19]. In many plants, the abscission layer is formed long before the activation of cell separation and breakage occur [19,20]. Seed shattering in *Oryza *is dependent on the proper formation and subsequent degradation of an abscission layer between the flower and the pedicel. QTL (quantitative trait loci) associated with loss of shattering have been identified on nearly every rice chromosome, and three loci have been cloned to date: *sh4/SHA1*, *qsh1 *and *OsCPL1 *[8,21,22]. Of these loci, *sh4*, which encodes a nuclear transcription factor, is considered the most important contributor to reduced shattering during rice domestication [23]. A single nonsynonymous substitution (G to T) in the first exon of *sh4 *leads to reduced function of SH4 and incomplete development of the abscission layer in non-shattering cultivated rice [8]. This non-shattering mutation is fixed in all cultivated rice varieties examined to date [8,18,24,25], spanning the highly differentiated *japonica *and *indica *cultivar groups. There is still some controversy whether Asian rice was independently domesticated at least twice from *O. rufipogon *populations [26-28], or only once [3,29]. Regardless of the domestication scenario, the ubiquity of the T substitution in cultivated rice suggests very strong selection for loss of shattering (perhaps in combination with introgression) during domestication [8,24,25].

Recently, we examined the seed shattering phenotype and the *sh4 *shattering locus in populations of U.S. weedy rice [18]. Several genetically differentiated populations of weedy rice occur in the U.S., and these can be distinguished by their predominant hull morphology [30]. Main populations include the straw-hulled (SH) group, early flowering weeds characterized by straw-colored hulls and lack of awns, and the black-hulled awned (BHA) group, later flowering weeds with seeds that have predominantly black hulls and long awns [30-32]. Genome-wide data indicate that SH and BHA weedy rice groups share genomic identity with Asian domesticated rice from the *indica *and *aus *variety groups, respectively, suggesting weedy origins within these cultivated groups [30,32,33]. Minor U.S. weedy rice groups include the brown-hulled (BRH) group, which are putative hybrids between SH and BHA weeds, and the mixed groups (MX), containing individuals likely to be hybrids between weeds and local *tropical japonica *cultivars [30]. We have found that nearly all U.S. weedy rice readily shatters its seeds to a similar degree as wild rice [18]. However, all populations of U.S. weedy rice share the "non-shattering" *sh4 *substitution common to cultivated rice, regardless of their propensity to shatter [18]. These results support the evolution of U.S. weedy rice from cultivated ancestors and, since wild and major weedy groups have separate origins, the parallel evolution of the shattering trait among these *Oryza *groups. Our results further imply that weedy rice re-acquired the shattering trait through the involvement of unidentified loci other than *sh4 *[18].

In an effort to understand how weedy rice may have re-evolved the shattering trait after its loss in domesticated ancestors, we investigate here the morphological basis of shattering in U.S. weedy rice groups. Given that wild and weedy rice do not share the ancestral *sh4 *shattering substitution characteristic of *O. rufipogon*, it is possible that wild and weedy groups do not share the same morphological shattering mechanism. Moreover, despite sharing the same "non-shattering" mutation at the *sh4 *locus [18], the two major U.S. weedy rice populations -- SH and BHA -- have separate origins, and may have acquired the shattering phenotype in mechanistically different ways, representing a separate instance of parallel evolution. To our knowledge, no study to date has investigated the morphological basis of the shattering trait in weedy rice. We examine the abscission layer at the flower-pedicel junction in weedy rice prior to, at and shortly after flowering to determine morphology and level of degradation of this layer in relation to seed shattering ability, and compare these results to those of wild and cultivated *Oryza*, to gain insight into how traits important to weed fitness can evolve.

## Results and Discussion

### Abscission Layer Formation Differs in Wild and Cultivated *Oryza*

**Figure 1:**
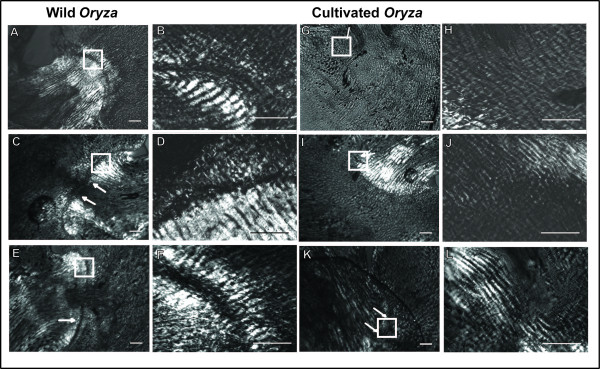
**Comparison of wild and cultivated *Oryza *flower-pedicel junctions**. Panels A-F are wild *Oryza *(A/B- 2F02 (*O. nivara*), C/D- 2F01 (*O. nivara*), E/F- 2C02 (*O. rufipogon*)). Panels G-L are cultivated *O. sativa *varieties (G/H- 3A11 (*indica*), I/J- 3A06 (*aus*), K/L- 3A08 (*indica*)). Arrows point to the region of the abscission zone, while white boxes show the region magnified further at right. Abscission layers can be seen as darkly stained bands. All samples shown here were taken at flowering for their respective accession and are all magnified at 10× on the left and 60× on the right. Scale bars on bottom right represent 100 μm for 10× images and 50 μm for 60× images.

We observed the abscission layer at the flower-pedicel junction at flowering in six wild *Oryza *(Table 1, donated with asterisk): four *O. rufipogon*, the wild ancestor of cultivated Asian rice, and two *O. nivara*, an annual ecotype of *O. rufipogon *[34]. All six wild *Oryza *show clear abscission layers between the flower and the pedicel at flowering (Figure 1A-F, and data not shown). The layer is slightly curved and occurs on both sides of the vascular bundle. Further magnification (60x) of the abscission layer shows very dark staining of cells at the center of the layer with some cells beginning to swell. This dark staining is most likely due to high lignification of these cells' walls, as abscission layer cells have been shown previously to be highly lignified [35]. Cells surrounding the layer are highly organized into rows and perpendicular to the plane of abscission. (Figure 1B, D, F). No degradation of the abscission layer is yet observed at this stage. The occurrence of well-developed abscission layers upon flowering suggests that all six wild *Oryza *accessions will shatter their seeds readily, an observation that is consistent with our previous measurement of shattering levels of ripe seeds in these accessions (average Breaking Tensile Strength (BTS) = 0 g, Table 1; also see [18]).

**Table 1:** List of Accessions used for this study.

Group	**Study ID **^**a**^	**USDA ID/Common Name **^**c**^	IRGC/RA/GRIN	**Origin **^**b**^	**Mean BTS (gram) **^**d**^	Std. Dev
Weedy rice	SH_1A08*	1134-01	x	AR	0	0
	SH_1A09*	1135-01	x	AR	0.3	0.5
	SH_1C02*	1001-01	x	AR	1	2
	MXSH_1B06*	1996-01	x	AR	35.6	17.9
	BHA1_1B08*	1996-09	x	MS	7.2	21.6
	BHA1_1A05*	1096-01	x	AR	0	0
	BHA1_1B02	10A	x	AR	0	0
	BHA1_1C04	1005-02	x	AR	0	0

Cultivated rice						
*aus*	3A06*	BJ-1	RA5345/45195	India	18.3	3.1
	2B03	Aus 196	29016	Bangladesh	12.3	9.8
*indica*	3C05	Dee_Geo_Woo_Gen	RA5344/PI279131	Taiwan	60.9	25.3
	3A11*	Dholi Boro	RA4984/27513	Bangladesh	137.4	11.8
	3A08*	Rathuwee	RA4911/8952/PI584605	Sri Lanka	72.3	47.8
	2B02	Bei Khe	22739	Cambodia	30.1	17.5
	3A09*	Khao Dawk Mali -105	RA4878/27748	Thailand	80.7	42.6
*tropical japonica*	3B09	Mirti	RA4970/25901/PI584553	Bangladesh	12	22.9
	3B12	Gotak_Gatik	RA4959/43397/PI584572	Indonesia	104.5	67.7

Wild Asian rice						
*O. rufipogon*	2C02*	N/A	100588	Taiwan	0	0
	2C09	N/A	104833	Thailand	0	0
	2C04	N/A	100916	China	0	0
	2C12	N/A	105491	Malaysia	0	0
	2D06*	N/A	106086	India	0	0
	2D12*	N/A	106169	Vietnam	0	0
	2E01*	N/A	106321	Cambodia	0	0
*O. nivara*	2F01*	N/A	86662	Thailand	0	0
	2F02*	N/A	103821	China	0	0

a Based on STRUCTURE and identity from Reagon et al, 2010

b Origin for weeds is a U.S. state abbreviation, origins for cultivated and wild rice is country

c Accessions with RA numbers were acquired from Susan McCouch while all others were acquired from IRRI, these ID's were also used in Reagon et al, 2010.

d BTS (Breaking Tensile Strength) corresponds to the maximum weight a seed can hold before releasing; from data reported in Thurber et al, 2010

*-- Individuals used for Microscopy; all others used only for shattering time course

x-- no data available

We also observed the flower-pedicel junction at flowering in four cultivated rice samples (Figure 1G-L and data not shown) belonging to the *aus *and *indica *cultivar groups, the putative ancestors of U.S. weedy rice. None of the spikelets (i.e. rice flowers with attached glumes) sampled shows formation of a clear abscission layer upon flowering, although two *indica *accessions (3A09 and 3A11; Figure 1G, H, K, L) show weak staining in the region of the abscission layer. In these accessions, further magnification shows diffuse staining of cells in the abscission zone, although cellular organization is not as defined as in the wild tissue samples at this stage (Figure 1H, J, L). This further supports the absence of an abscission layer, and, in all cultivated samples, the pedicel blends in easily with the floral tissue at flowering. The lack of an abscission layer at flowering in all three *indica *cultivated accessions is consistent with their lack of shattering (average BTS = 70 to 137 g, Table 1). The single *aus *sampled is considered a very easy seed releasing variety (average BTS = 18 g, Table 1), yet it also appears to not possess an abscission layer at flowering (Figure 1G, H), suggesting that formation of this layer may be delayed and incomplete.

Our overall observations of clear abscission layers upon flowering in shattering wild *Oryza *individuals and lack of abscission layers at this stage in non-shattering cultivated rice are consistent with previous studies (see [8,17,21,25]), and serve as a baseline for comparison to weedy rice. Because our observations do not differ from those published previously for other cultivated and wild rice samples, we concluded that abscission layer traits are robust under our growth conditions, and we did not sample additional time points of abscission layer development. Studies have documented that the abscission layer begins to form at least one week prior to flowering in wild *O. rufipogon *(and some exceptionally easy threshing *indica *and *aus *cultivars), and by flowering is prominent and clearly visible with staining [25,36-39]. The abscission layer in *O. rufipogon *begins to degrade at or within a week of pollination, about two weeks after flowering, and continues degradation as the seed begins to form and mature, until the seed is released [37-39]. In contrast, in cultivated rice varieties, the abscission layer (if present) remains intact for at least 12 days after pollination [25]. Both previous studies and ours show that there are dramatic differences in abscission layer formation and degradation between wild and cultivated rice, likely due to selection against shattering during the domestication process.

### Degradation of the Abscission Layer is Accelerated in Weedy Rice

To determine the role of abscission layer formation and degradation in the shattering phenotype of weedy rice, we sampled six weedy rice accessions from three separate groups (SH (3), BHA (2), MX (1); Table 1, denoted with asterisk) at each of three time points: prior to, at and after flowering. With the exception of the non-shattering MX accession (MXSH_1B06, average BTS = 35 g, Table 1), all other weedy rice shatter easily, regardless of population identity (average BTS < 8 g, Table 1). We chose the single MX individual, as it was the only accession found in [18] that did not shatter extensively, and was one of the few accessions identified as a putative hybrid between SH weeds and U.S. *tropical japonica *[30]. We hypothesized that abscission layer formation and degradation in shattering weedy samples would resemble that observed for *O. rufipogon *and *O. nivara*, while the non-shattering weed individual would resemble cultivated rice.

**Figure 2:**
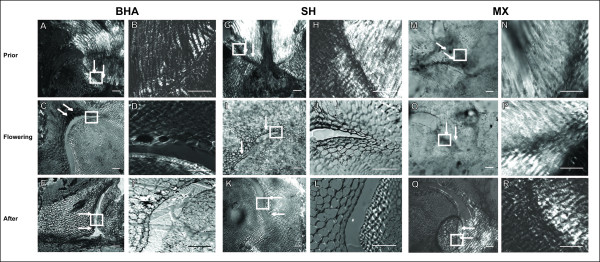
**Comparison of abscission layers across weedy *Oryza *populations**. Panels A-F are shattering BHA_1A05, Panels G-L are shattering SH_1A08, Panels M-R are non-shattering MXSH_1B06. Each individual was collected 1 week prior to flowering (Prior), at flowering (Flowering) and 1 week after flowering (After). Arrows point to the region of the abscission zone while white boxes outline the region magnified further. Abscission layers can be seen as darkly stained bands. Images at left were taken at 10× magnification while those at right are 60× magnification. Scale bars on bottom right represent 100 μm for 10× images and 50 μm for 60× images.

One week prior to flowering, all five shattering weedy rice accessions, including the two shown in Figure 2 (SH_1A08 and BHA_1A05) possess well-defined abscission layers (Figure 2A, G). Inspection with a higher magnification 60× lens shows that the BHA and SH weedy rice abscission layers prior to flowering (Figure 2B, H) are similar in staining and organization to the wild rice at flowering stage (Figure 1B, D, F); the highly lignified cells are darkly stained and starting to swell slightly, while the cells around the region are parallel to the plane of abscission. In contrast, the non-shattering MX weed shows only unbalanced, diffuse staining in the abscission zone with no clear organization of cells surrounding the zone (Figure 2M, N).

At flowering, the abscission layers for all the BHA and SH shattering weeds already show mild to moderate degradation and swollen cells at the abscission zone (Figure 2C, I; Additional File 1). Further magnified images show very swollen cells at the abscission layer with the darkest staining seen on the edges that are now exposed due to breakage (Figure 2D, J). All five shattering weeds already show degradation that is not observed in their shattering wild relatives at the flowering stage, yet there is some variation in the degree of degradation between weed accessions (Figure 1; Additional File 1). In contrast, the non shattering MX still shows only diffuse, weak staining, yet is beginning to form an abscission layer to one side of the vascular bundle (Figure 2O, P). Interestingly, when compared to wild and cultivated spikelets at this developmental stage, MX looks very similar to the non-shattering *indica *cultivars (Figure 1G, I, K).

A week after flowering has occurred, which is roughly one to two weeks prior to seed set in weedy rice, all SH and BHA shattering weeds sampled show moderate to near complete separation at the abscission layer and are only held together at the tips of the layer and the vascular bundle (Figure 2E, K, and data not shown). Cells that are still attached at the layer are swollen and darkly stained along the plane of breakage. Cells that have already been separated are losing their dark staining, possibly due to rearrangement of cell wall components (Figure 2F, L). A week after flowering, the non-shattering MX individual has developed a complete abscission layer, yet the cells at this layer have not begun to swell or degrade (Figure 2Q). When examined more closely, the cells of the non-shattering weed look very similar to wild abscission layer cells at flowering and to the shattering weeds prior to flowering: the cells are darkly stained and show a clear abscission layer with organized cells in the abscission zone (Figure 2R).

Taken together, our microscopy results demonstrate that shattering weeds display abscission layer developmental differences compared to wild and cultivated rice. Both wild and weedy individuals develop similar looking abscission layers in the same location of the floral-pedicel junction; this similar cellular morphology is consistent with the shared shattering trait of wild and weedy individuals. Moreover, abscission layer formation in shattering weedy rice occurs at least one week prior to flowering, if not earlier, similar to what has been reported for shattering wild rice [25,36]. However, at flowering, the abscission layer in weedy rice has already begun to degrade, in some cases severely, which is not the case in shattering wild rice or easy threshing varieties of cultivated rice [17] (Figures 1 and 2; Additional File 1). This suggests that timing of abscission layer degradation, rather than morphological differences, distinguishes the shattering trait in weedy and wild rice groups. Surprisingly, despite their independent origins from separate cultivar groups (*aus *and *indica*, respectively), both BHA and SH weeds show similar abscission layer traits and timing. This suggests that both U.S. weedy rice groups may have re-acquired the shattering trait in a similar mechanistic manner, opening the question of whether common genetic elements are involved.

Further investigation of additional developmental stages and a finer scale of developmental series may help identify more precisely when the abscission layer forms in weedy rice and how rapidly after formation it degrades. It is unclear from previous studies how the abscission layer degradation process is activated in rice, yet it is possible that the degradation repertoire is activated only after a certain stage of abscission layer development is complete. While further research is needed, our results indicate that weedy rice may reach this formative stage earlier than wild shattering relatives, and as a result, show earlier degradation. It is also possible that the formation of the abscission layer progresses at the same rate in both weedy and wild rice, with weedy rice abscission activating their degradation repertoire earlier in abscission layer formation than in wild rice.

### Seed Shattering Time Course Profiles are Altered in Weedy Rice Compared to the Wild Relatives

The early degradation of U.S. weedy rice abscission layers may confer an earlier shattering phenotype than reported for wild rice. Earlier degradation of the abscission layer suggests that as soon as the weedy seed is mature, or nearly so, it can more readily fall to the ground. The timing of seed release is considered important to weed fitness, as it may be beneficial to disperse seeds prior to harvest [40]; earlier shattering could thus be a response to rice cultivation practices. Additionally, or alternatively, earlier release may prevent seeds from drying out and losing dormancy, another trait that enhances weediness [41]; higher moisture content in seeds is known to confer a greater level of dormancy [42], but desiccation of rice seeds occurs as they mature. Easy shattering may not necessarily always be an advantage, however. Seeds that shatter before they are mature enough to germinate will lower a plant's fitness [36].

**Figure 3:**
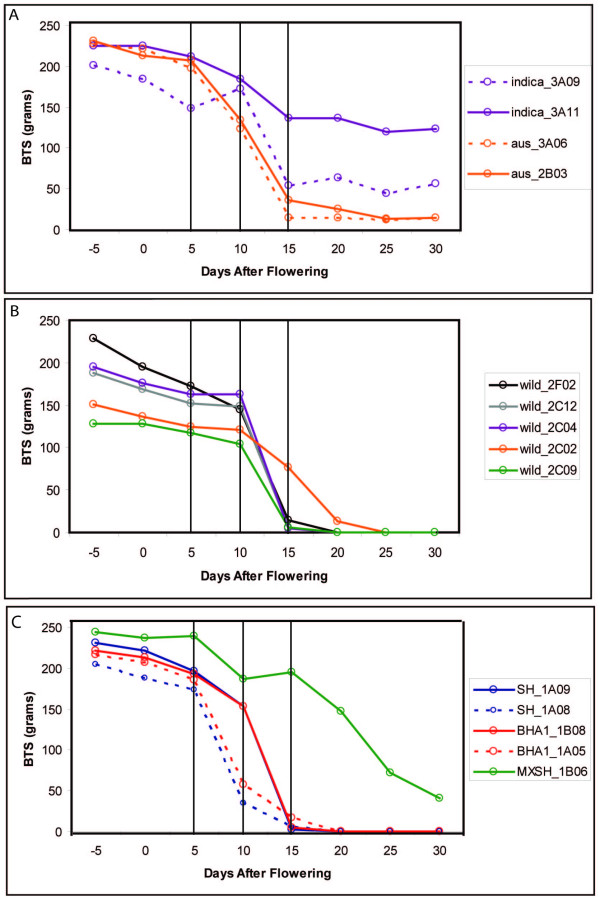
**Shattering across floral and grain development**. Shattering levels for cultivated (4), wild (5) and weedy (5) individuals were recorded every five days from 5 days prior to flowering (-5) through 30 days after flowering (30). Panel A shows shattering levels for cultivated rice, Panel B shows shattering levels for wild rice, and Panel C shows shattering levels for weedy rice.

Phenotypically, little is known about the shattering levels in weedy rice groups across floral/seed development. Previous studies in cultivated and wild rice have shown that shattering level increases dramatically after 15 days post flowering in wild rice and in some cultivated rice samples grown in both field and greenhouse settings [17,36]. In an effort to determine if shattering levels mirror the observed formation and degradation of the abscission layer in U.S. weedy rice groups, we assessed levels of shattering as the amount of weight a grain can hold prior to release from the panicle (breaking tensile strength; BTS) in eight cultivated, five wild and seven weedy rice individuals, at various time points through seed development (Figure 3 and Additional File 2).

To date, we have examined eight cultivated rice varieties from the *indica*, *aus *and *tropical japonica *groups (Additional File 2). Four of these samples are shown in Figure 3A (3A06, 3A11, 2B03 and 3A09). All cultivated rice accessions show consistent high BTS values between 150 g to 250 g from before flowering through ten days after flowering. By 15 days after flowering, BTS values have dropped close to the level previously seen in these cultivars at maturity (between 25 g and 125 g), and remain at these levels through 30 days after flowering, consistent with measurements reported in [18]. The five wild rice individuals surveyed (2F02, 2C12, 2C04, 2C02 and 2C09) show a similar shattering pattern to cultivated rice up through ten days post flowering (Figure 3B and Additional File 2). However, at 15 days post flowering, the BTS levels have dropped dramatically to near 0 g and stay at this level through 30 days post flowering (Figure 3B and Additional File 2). This is consistent with all reported observations of *O. rufipogon *and *O. nivara *shattering behavior across floral development [17,36], and is consistent with the wild rice seed shattering trait at maturity (Table 1).

All six shattering weeds examined (SH_1A08, SH_1A09, BHA1_1B08, BHA1_1A05, BHA1_1C04 and BHA1_1B02) registered BTS values above 150 g five days before through five days after flowering (Figure 3C and Additional File 2). By ten days after flowering, BTS values for three weeds (SH_1A08, BHA1_1C04 and BHA1_1A05) have dropped to below 60 g, while all other weeds are still registering values around 150 g. By fifteen days after flowering, all shattering weeds shown have dropped their BTS values dramatically to nearly 0 g (Figure 3C and Additional File 2). The BTS values thereafter stay at 0 g throughout the remainder of seed maturation for all shattering weeds shown. The single non-shattering weed (MXSH_1B06) shows a different time course as the shattering weeds. The sharpest decreases in BTS values are only seen after 20 days after flowering and instead of dropping to 0 g the BTS values for this individual only go as low as 40 g (Figure 3C and Additional File 2).

The variation in timing of the sharp reduction in BTS values across the weeds surveyed indicates that shattering ability is only partly correlated with abscission layer degradation rates. Though all weedy rice accessions used in our microscopy study displayed earlier degradation of the abscission layer than what is seen in wild rice, a range of degradation severity seems to exist (Figure 2; Additional File 1). Two weed samples that showed reduction in BTS values five days prior to other weeds tested appear to possess the highest degraded abscission layers at flowering (Figure 2). Weeds with drastically reduced BTS values at 15 days, a timing consistent with that of wild rice, seem to have somewhat less-degraded layers at flowering (Additional File 1). Overall the weedy rice individuals that showed the least degradation at flowering have similar shattering time courses to what has been shown previously for wild rice, while those with the most degradation show an earlier drop in BTS values. This indicates that the timing of when shattering is first noticeable in weedy rice is variable, despite the fact that all weeds degrade their abscission layer at an earlier time than wild rice.

### Novel mutations likely underlie the parallel evolution of shattering in weedy and wild rice

Previous studies of the *sh4 *locus in wild and domesticated rice have implicated this gene in both the formation and degradation of the abscission layer at the flower-pedicel junction [8,25]. A mutation in the *sh4 *gene, strongly selected upon during rice domestication, is associated with reduction in shattering in cultivated rice varieties due to the formation of a discontinuous abscission layer [8]. Transgenic experiments have further demonstrated that the ancestral *sh4 *allele (present in wild *O. rufipogon*) can restore shattering in non-shattering cultivated rice [8]. Our previous work showed that U.S. weedy rice groups carry the derived non-shattering mutation fixed in cultivated rice [18], demonstrating that the functional mutation identified in the *sh4 *locus does not result in non-shattering in the weed, and is thus not sufficient for loss of shattering. This suggested that novel loci, perhaps distinct from those acting in wild rice species, are involved in the evolution of shattering in U.S. weedy rice groups.

The distinct developmental profile observed here for weedy rice abscission layers further supports that U.S. weedy rice groups did not acquire the shattering trait through introgression with wild species. Thus, this and our previous work [18] suggest that parallel evolution of shattering in weedy and wild rice has occurred through both different loci and different developmental mechanisms. Studies in several other systems have shown that parallel evolution between populations can arise from independent mutations in the same gene, as has been shown for body shape characteristics in two independent populations of freshwater stickleback and for two independently evolved populations of melanic *Peromyscus *rodents [43,44]. Conversely, studies of independent melanic populations of rock pocket mice have also shown that convergent phenotypes can sometimes be achieved through mutations in different genes [45,46]. The acquisition of the shattering trait in wild and weedy rice groups further supports the possible role of independent loci in parallel evolution.

Interestingly, the similarities in abscission layer traits (development and shattering time course) between two distinct weedy rice groups, SH and BHA, suggest that the gene(s) involved in reacquiring seed shattering may be the same in both populations. This is surprising, as these groups have been shown to have independent evolutionary origins [30,32]. The convergence in the mechanistic basis of seed shattering among these weedy rice groups may indicate certain genetic or morphological constraints inherent to re-evolving the shattering trait after its loss through domestication. Future studies into the genes involved in the progression of abscission layer formation and degradation in both weedy and wild rice will be integral to the study of weed evolution.

## Conclusions

Our results show that the shattering trait in U.S. weedy rice has a distinct mechanistic basis from that of the shattering wild ancestor of rice, consistent with the re-evolution of this trait in weedy groups from domesticated ancestors. Surprisingly, independently evolved weedy groups have converged on this feature of abscission layer development. In some cases, the altered timing of abscission layer degradation appears to lead to earlier shattering in weedy rice compared to wild rice.

## Methods

### Plant materials for microscopy

All accessions used in this study are a subset of those used in [18] for which phenotypic and sequence data are available. Five weedy rice accessions, collected in the Southern U.S. rice belt, were generously supplied by David Gealy (USDA) (Table 1). Accessions were chosen to represent the two major weedy rice groups (SH and BHA) based on population structure analysis [30] and a group of putative weed-crop hybrids (MX) showing some resistance to seed shattering. Additional samples of wild and cultivated *Oryza *were originally obtained from the International Rice Research Institute (IRRI) (*O. rufipogon *(4) and *O. nivara*, a close relative or annual ecotype of *O. rufipogon *(2)) and Susan McCouch (*O. sativa *(4)). All plants were grown in a Conviron PGW36 growth chamber at the University of Massachusetts Amherst. One seed per accession was planted in a 4 inch pot and grown as described in [18]. Panicles from wild and cultivated individuals were collected at flowering, while panicles from weedy individuals were harvested at three time points: one week prior to flowering, at flowering and one week after flowering. For observations prior to flowering, panicles were collected when the boot, or flag leaf sheath, was swollen yet before flowers had begun emerging. At flowering, panicles were collected once 50% of the panicle had emerged from the boot. Panicles to be collected after flowering were bagged upon flowering to prevent pollen flow and loss of seeds. At each collection, approximately eight flower-pedicel tissue samples were excised from the flowers at the topmost end of the panicle using a dissecting scope.

### Microscopy

Tissue samples were fixed with glutaraldehyde (100 mM) in a solution containing 100 mM PIPES pH 7.0, 100 mM Glutaraldehyde, 0.5 mM CaCl_2_, and 5.0 mM MgCl_2 _for 2 hours. Following fixation samples were dehydrated first in an ethanol series then further dehydrated in acetone. Dehydrated samples were infiltrated and embedded in Epon Araldite resin [47]. Samples were sectioned longitudinally using a diamond knife on a rotary microtome (Porter-Blum JB4) to create 2 micrometer sections. Sections were dried onto rectangular microscope slides and subsequently stained for 3 minutes with Toluidine Blue (0.5% solution in 0.1% sodium carbonate, pH 11.1), a metachromatic dye which stains regions with high lignin dark blue-green and regions of unlignified cell wall reddish purple (see [48]). Bright field images were taken at both 10× and 60× using a Nikon TE 300 Inverted Microscope with an attached CCD camera (Quantix CoolSnap HQ; Roper Scientific).

### Time course shattering measurements

Five weedy rice accessions, along with five wild rice accessions and eight cultivated *O. sativa *accessions (see above) were analyzed for shattering ability during floral and seed development (Table 1). All plants were grown as described above for microscopy. Panicles from each individual were collected ~5 days before flowering (swollen boot with top most flower of panicle approaching emergence), at flowering (50% of panicle emerged from boot), as well as 5, 10, 15, 20, 25, and 30 days after flowering. Upon flowering, panicles to be collected were bagged to prevent pollen flow and loss of seeds. The oldest (topmost) 10 flowers per panicle were analyzed for breaking tensile strength (BTS), or shattering level, using a digital force gauge as described in [18]. BTS is a measure of the maximum amount of weight, in grams, a single flower or grain can hold before releasing; values at or near zero grams (g) are considered highly shattering while values over 100 g represent non-shattering or hard threshing [8,18,21]. Average BTS values for the ten measurements are reported for each sample.

Accessions are identical to those used in a previous study [18] and are grouped by type (weed, wild or cultivar). Identification numbers as well as phenotypic values for seed shattering are reported here as well as in [18].

## Authors' contributions

ALC and CST conceived the study. CST and PKH carried out the microscopy. CST carried out the time course shattering experiments. ALC and CST wrote the paper. All authors read and approved the final manuscript.

## Author's information

This work is part of CST's PhD thesis research into parallel evolution of weed traits in crop weeds.

## Supplementary Material

Additional File 1 **Additional weedy rice abscission layer images at flowering**. Samples shown here were taken at flowering for their respective accession and are all magnified at 10× with scale bars on bottom right representing 100 μm. Arrows point to the breakdown of the abscission layer. Click here for file

Additional File 2 **Average BTS values across floral and grain development**. Average BTS values for each individual at -5. 0, 5, 10, 15, 20, 25 and 30 days after flowering, recorded in grams. Click here for file
