# Supplementary figures and images for: Timing is everything: early degradation of abscission layer is associated with increased seed shattering in U.S. weedy rice
Source: BMC Plant Biol. 2011 Jan 14;11:14. doi: 10.1186/1471-2229-11-14 (PMC3025945; doi:10.1186/1471-2229-11-14)

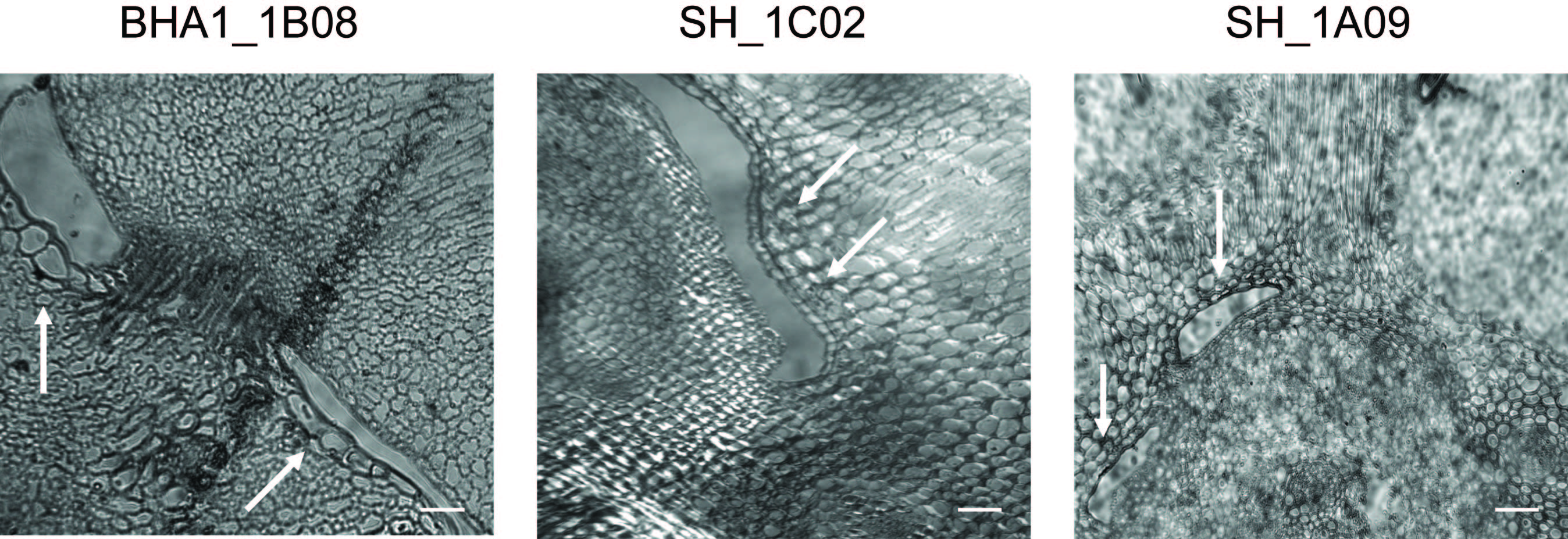

Supplement: Additional File 1 — Additional weedy rice abscission layer images at flowering. Samples shown here were taken at flowering for their respective accession and are all magnified at 10× with scale bars on bottom right representing 100 μm. Arrows point to the breakdown of the abscission layer. [file 1471-2229-11-14-S1.JPEG]
